# High cognitive reserve is associated with a reduced age-related deficit in spatial conflict resolution

**DOI:** 10.3389/fnhum.2012.00327

**Published:** 2012-12-12

**Authors:** Olga Puccioni, Antonino Vallesi

**Affiliations:** ^1^Neuroscience Area, International School for Advanced Studies (SISSA)Trieste, Italy; ^2^Department of Neurosciences: Neurological, Psychiatric, Sensorial, Reconstructive, and Rehabilitative Sciences, University of PadovaPadova, Italy

**Keywords:** cognitive aging, conflict, cognitive reserve, spatial Stroop

## Abstract

Several studies support the existence of a specific age-related difficulty in suppressing potentially distracting information. The aim of the present study is to investigate whether spatial conflict resolution is selectively affected by aging. The way aging affects individuals could be modulated by many factors determined by the socieconomic status: we investigated whether factors such as cognitive reserve (CR) and years of education may play a compensatory role against age-related deficits in the spatial domain. A spatial Stroop task with no feature repetitions was administered to a sample of 17 non-demented older adults (69–79 years-old) and 18 younger controls (18–34 years-old) matched for gender and years of education. The two age groups were also administered with measures of intelligence and CR. The overall spatial Stroop effect did not differ according to age, neither for speed nor for accuracy. The two age groups equally showed sequential effects for congruent trials: reduced response times (RTs) if another congruent trial preceded them, and accuracy at ceiling. For incongruent trials, older adults, but not younger controls, were influenced by congruency of trial_*n*−1_, since RTs increased with preceding congruent trials. Interestingly, such an age-related modulation negatively correlated with CR. These findings suggest that spatial conflict resolution in aging is predominantly affected by general slowing, rather than by a more specific deficit. However, a high level of CR seems to play a compensatory role for both factors.

## Introduction

Everyday several kinds of information are simultaneously processed by our brain, and one of the most demanding tasks is to cope with conflicting incoming stimuli. For example, crossing an intersection when the traffic light is green does not require a high level of attention, but it is essential that our attentional system is able to increase control when needed, such as when a traffic officer is directing your car to stop despite the green light. In this second case, you must inhibit the more automatic response in favor of the non-routine action. Since avoiding critical errors by suppressing inappropriate, but prepotent responses is crucial in everyday life, conflict resolution is a widely studied aspect of selective attention.

Different aspects of conflict have been investigated, but a well-known type of conflict is the semantic one tapped in the classic color-word Stroop task. It is still a matter of debate whether there is a general system to cope with cognitive conflict, devoted to the resolution of conflict, rather than multiple domain-specific ones. In the classic color-word Stroop task, participants are required to respond to the ink color (target) of a word and to ignore the word meaning (distractor), itself referring to a color (e.g., RED written in blue). This task has been widely used in order to explore verbal conflict resolution (Stroop, 1935; Dyer, 1973; Posner and Snyder, 1975; MacLeod, 1991), whereas its spatial version has been used less frequently (e.g., Lu and Proctor, 1995; Funes et al., 2010). In the spatial version of the Stroop task, the stimulus is usually an arrow positioned in a specific position on the screen. The task consists of responding according to the direction of the arrow (target) while ignoring its position (distractor). If the distractor and the target refer to the same spatial feature, the stimulus is considered “congruent,” whereas when they refer to different spatial features conflict takes place, and the stimulus is therefore called “incongruent”. Conflict is usually measured as the Stroop effect, that is the performance difference between congruent (C), or conflict absent, and incongruent (I), or conflict present, trials.

Moreover, conflict sequential effects have been also reported in many different tasks, such as the color-word Stroop (e.g., Kerns et al., 2004), the Simon (Strümer et al., 2002; Wühr, 2005; Kunde and Wühr, 2006; Notebaert et al., 2006; Vallesi and Umiltà, 2009), and the flanker (Gratton et al., 1992) tasks. These sequential effects consist on the fact that the congruency of the preceding trial (trial_*n* − 1_) influences conflict resolution in the current trial (trial_*n*_): the Stroop effect decreases after incongruent trials_*n* − 1_ and increases after congruent trials_*n* − 1_. This well-known pattern of conflict adaptation, also known as Gratton effect (Gratton et al., 1992; Kerns et al., 2004; West and Moore, 2005), suggested the presence of a conflict monitoring system, which includes the anterior cingulate and lateral prefrontal cortex (Botvinick et al., 2001). This system increases cognitive control whenever conflict is detected, in order to reduce the influence of distracting information. An alternative set of theories suggests that such a conflict adaptation pattern is due to priming or binding effects, rather than to a specific monitoring/resolution system. In fact, the subsequent presentation of stimuli with the same type of congruency increases the probability that one or both features constituting the stimulus would also be repeated, giving rise to negative and repetition priming (Mayr et al., 2003; Nieuwenhuis et al., 2006), or to binding (e.g., Hommel et al., 2004; Notebaert and Verguts, 2007) phenomena.

The prefrontal cortex plays an important role in conflict resolution (Botvinick et al., 2001; Mansouri et al., 2007; Floden et al., 2011), and since many cognitive aging theories assume a progressive decline of frontal brain areas (e.g., West, 1996; Braver and Barch, 2002; MacPherson et al., 2002), it is not surprising that many authors reported an age-related decrease in conflict resolution abilities, both in color Stroop tasks (e.g., West and Bell, 1997; West and Alain, 2000; Davidson et al., 2003) and in spatial Stroop or Simon tasks (Van der Lubbe et al., 2002; Bialystok et al., 2004; Proctor et al., 2005; Castel et al., 2007). Indeed, West's “frontal lobe hypothesis” of cognitive aging (West, 1996) asserts that cognitive processes supported by the prefrontal cortex are more prone to age-related decline with respect to processes subtended by non-frontal regions (Dempster, 1992; Moscovitch and Winocur, 1992; Hartley, 1993; Juncos-Rabadán et al., 2008). On the other hand, since an increase of response times (RTs) is consistently found in cognitive aging studies, a general slowing theory has also been proposed (Salthouse and Babcock, 1991; Salthouse, 1996), which suggests that non-pathological aging exerts a global slowing effect on cognitive processes. It is important to note that system-wide slowing and frontal lobe-specific declines can coexist. Therefore, in aging studies it is important to clarify whether older adults show increased RTs (1), because they are generally slower with respect to younger adults, (2) because aging has a specific impact on a well-defined deficit, or (3) because there is a combination of the two.

A number of previous studies about conflict resolution in aging drew their conclusions without applying a correction for general slowing. Furthermore, to the best of our knowledge, none of them properly separated the spatial conflict resolution process from priming/binding phenomena, which, as expressed above, could be largely responsible for the observed conflict adaptation effects. The main aim of the present study is to verify whether there is an age-related deficit specific for spatial conflict resolution, after controlling for the possible confounds due to priming or binding effects. We addressed this issue by using a priming-free spatial Stroop design (at least in terms of first-order trial sequences), in order to minimise the contribution of repetition priming on conflict resolution.

Another goal of the study was to explore whether intelligence and some factors influenced by the socio-economic status, such as cognitive reserve (CR) and years of education might partially account for the age-related inter-individual variability in performance. Some studies suggest a strong relationship between intelligence and a high level of cognitive functioning in aging (e.g., Alexander et al., 1997; Albert and Teresi, 1999). We therefore used a subset of the Wechsler Adult Intelligence Scale subtests (WAIS; Wechsler, 1981) in order to assess each participant's Intelligence Quotient (IQ) and correlate it with performance measures. In the course of the last few decades, the concept of CR (Stern et al., 1994, 1995) has been used to explain why similar brain damage could lead to different levels of cognitive impairment (Katzman et al., 1989; Ince, 2001). CR has been described as a set of beneficial effects on neural plasticity and cognitive strategies derived from a lifestyle rich of experiences, such as a higher education level, occupational attainment and leisure activities (Rocca et al., 1990; Evans et al., 1993; Stern et al., 1994, 1995; Mortel et al., 1995; Manly et al., 2003; Valenzuela and Sachdev, 2006). This approach suggests that a high level of CR may be a key factor to delay the decline of cognitive functions, in as much as the use of pre-existing cognitive processes, or the development of new compensatory processes, could allow the aging brain to cope actively with loss of functionality (Stern, 2002). Indeed, many studies have suggested a compensatory role of CR against non-pathological cognitive aging (e.g., Evans et al., 1993; Stern, 2002).

Intelligence and CR might have distinct compensatory impact on cognitive functioning (Stern, 2002, 2009). Moreover, it is always necessary to be careful with data that relate intelligence and cognitive aging, since it is difficult to separate the expected beneficial effects of high IQ with respect to a possible compensation.

As Nucci et al. (2012) pointed out, intelligence definition and measurement are based on intellectual performance (Wechsler, 1944), whereas cognitive reserve is based on the concept of cognitive skills acquired during one's lifetime (e.g., Stern, 2002, 2009). Therefore, we decided to measure and treat cognitive reserve and intelligence separately. We anticipate here that the absence of a significant correlation between CR and IQ in our sample (see Table 2) supports our assumption.

We assessed verbal and performance intelligence with a set of WAIS subtests (Wechsler, 1981), in addition to a measure of CR (Nucci et al., 2012, see section “Materials and Methods”). Additionally, we decided to consider years of education not only as a contribution for the CR score, but also as a separate independent predictor.

To summarize, we wanted to verify whether cognitive aging specifically affects spatial conflict resolution using a priming-free experimental task, similar to the one adopted in our previous works, which were focused on verbal conflict resolution (Puccioni and Vallesi, 2012, in press). We then analyzed our data by trying to disentangle between age-related general slowing and a specific conflict resolution deficit. Furthermore, this work aimed at investigating if factors such as CR, years of education and intelligence may play a compensatory role against age-related deficits in spatial conflict resolution.

## Materials and methods

### Participants

Seventeen older adults (mean age = 73 years, range 69–79; 8 females) and eighteen younger controls (mean age = 24 years, range 18–34; 9 females) participated in this study. All participants were native Italian-speakers and right-handed, as measured with the Edinburgh Handedness Inventory (Oldfield, 1971). All participants had normal or corrected-to-normal vision and normal color perception, as assessed with the Ishihara Color Vision Test (Ishihara, 1962). The two age groups had attained the same years of formal education on average (younger, range: 9–18, *M* = 13.4 years; older, range: 6–18, *M* = 12.5 years, [*t*_(33)_ = 0.764, *p* = 0.449]). None of the older adults met the criteria for dementia as assessed with the Montreal Cognitive Assessment (MoCA) (Nasreddine et al., 2005; score range: 26–30/30). Five of them reported regular consumption of medications for cardiovascular disease. Two extra participants were excluded: an older participant because of a low MoCA score (24/30), and a younger participant because of low intelligence scores (IQ = 72). The present study was approved by the SISSA ethical committee. Each participant signed an informed consent form and received 15 € to take part in the experiment. All participants also performed a verbal Stroop task in a different session, on a different day. The results of this other study are reported elsewhere (Puccioni and Vallesi, in press).

### Experimental material and design

Participants were individually tested in a dimly lit room, sitting at a distance of about 50 cm from the computer LCD monitor to perform the Stroop test. First, two single-feature tasks were administered: Direction Only, Position Only. Then participants were administered three spatial Stroop blocks. An instruction page preceded the presentation of each task.

**Figure 1:**
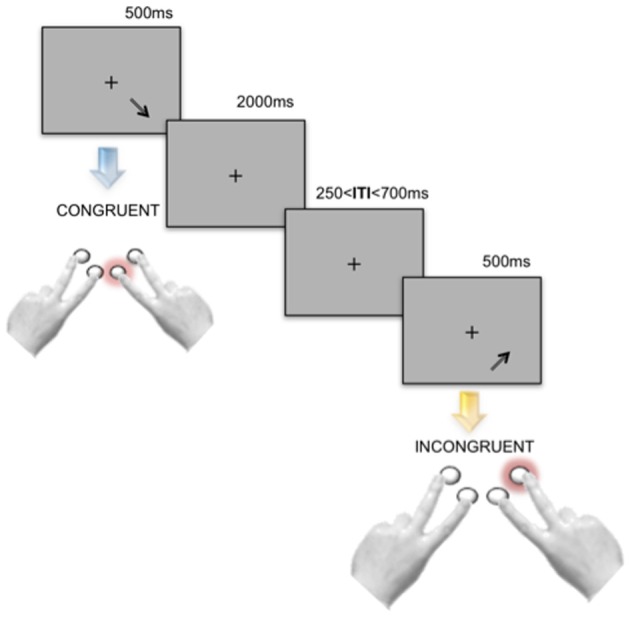
**Experimental design.** During all the three experimental tasks—Direction Only, Position Only and spatial Stroop—each stimulus was presented for 500 ms, followed by a blank screen showing the fixation cross and lasting 2000 ms. Before the onset of subsequent stimulus, an Inter-Trial-Interval (ITI) varying randomly and continuously between 250 and 700 ms was presented. Stroop stimuli were divided in Congruent and Incongruent with respect to the direction of the displayed arrow and its position on the screen. Participants were asked to respond by pressing one out of four response buttons displayed as shown in the panel. In the picture two Stroop trials are represented.

In each condition, the stimuli were presented against a light-gray background. Participants were asked to maintain their gaze on a fixation cross shown in the middle of the screen. Four response buttons were arranged in order to reflect the north–east, north–west, south–east or south–west directions (see Figure 1). Participants were asked to give a response as fast and accurately as possible by using the index and middle fingers of both hands.

In the Direction Only task, the fixation cross disappeared at stimulus onset and was replaced with one out of four different arrows (2.5 cm long × 1.5 cm wide) pointing to north–east, north–west, south–east or south–west. Participants were asked to indicate the direction of the arrow by pressing the corresponding button.

In the Position Only condition, stimuli were Xs (each arm 2.5 cm) appearing in one out of four different positions on the screen: upper right, upper left, lower right, lower left part of the screen (about 8 cm from the fixation cross). Participants were asked to indicate the position of each stimulus by pressing the corresponding button. For each of the two single-feature tasks, 32 test trials were administered, preceded by 2 training trials.

In the spatial Stroop task, one arrow out of four (pointing to north–east, north–west, south–east or south–west) appeared in one out of the four positions on the screen (upper right and left, lower right and left). Participants were required to ignore the position and to respond according to the pointing direction of the arrows by pressing the corresponding button.

The Stroop stimuli were categorized as *congruent* (e.g., north–east pointing arrow positioned in the upper right part of the screen) and *incongruent* (e.g., north–east pointing arrow positioned in the upper left part of the screen) and only complete alternation sequences were used. This implies that the position (distractor) and the direction (target) of the stimuli in trial_*n*_ were always different from their position and direction in trial_*n* − 1_ (e.g., north–east arrow in the lower right part, followed by north–west arrow in the lower left part). Moreover, we categorized subsequent pairs of trials according to the congruency status of trial_*n* − 1_ and trial_*n*_ as: congruent-congruent (CC), congruent-incongruent (CI), incongruent-congruent (IC), incongruent-incongruent (II). In order to familiarize with the task, participants performed a training phase at the beginning of the Stroop task, composed of 16 trials, with all possible position-direction combinations. During the training phase, each stimulus remained on the screen until a response was detected. Then feedback on accuracy and speed appeared for 1200 ms, followed by an Inter-Trial-Interval of 500 ms. The feedback was “Good” (in Italian: “Bene”), for a correct response within 2000 ms from trial onset; “Correct, but try to be faster … ” (in Italian: “Corretto, ma cerca di essere piú veloce … ”) for a correct response which occurred later than 2000 ms after stimulus onset; “Wrong” (“Sbagliato”) for incorrect responses. If participants made more than 6 errors out of 16 trials during the training phase, they had to repeat this phase. This was the case for five older adults, who had to repeat twice the training phase. The test phase was divided in three blocks, each one composed of 2 sub-blocks of 64 stimuli each. Each sub-block was composed of at least 30 *congruent* trials, in order to minimize the influence of task strategies-related to unbalanced frequencies of congruency conditions (Gratton et al., 1992; see also Vallesi, 2011). Each of the four possible sequential conditions (CC, CI, II, and IC) was presented in at least 15 trials (cf., Mordkoff, 2012). In each test trial, the target stimulus appeared at the center of the screen for 500 ms, followed by a blank of 2000 ms whose offset corresponded to the response deadline (2500 ms). An extra blank screen, which lasted randomly and continuously between 250 and 700 ms, was presented before the onset of the next trial.

Nine subtests of the WAIS-R (Wechsler, 1981) were administered to the participants in order to calculate their IQs. The selected subtests were: Block Design, Arithmetic, Vocabulary, Similarities, Comprehension, Digit Span, Digit-Symbol, Object Assembly, and Picture Completion. WAIS subtests were administered during the intervals between Stroop blocks in two separate sessions (counterbalanced order) run on different days. Younger and older adults samples resulted to have a comparable IQ [younger, range: 79–120, *M* = 100; older, range: 93–109, *M* = 100, (*t*_(33)_ = −0.03, *p* = 0.97)].

In order to quantify CR, after the last Stroop block older participants only were administered the “Cognitive Reserve Index questionnaire” (CRIq; Nucci et al., 2012). To compute the CR index, the contribution of factors such as activities (sport, leisure, and cultural), years of education, and occupation, carried out by participants during their adult lifetime were weighted and combined in a composite score (Nucci et al., 2012). As Nucci et al. point out, since there are no similar standardized instruments to measure this construct for the Italian population, it is not possible to assess concurrent validity of the CRIq (Nucci et al., 2012). Although this instrument presents some limitations, such as a modest reliability (Cronbach's α = 0.62, 95% Confidence Intervals: 0.56–0.97) (Nucci et al., 2012), we think that keeping intelligence and lifestyle-related factors separated is very interesting in order to investigate which aspect could modulate more the effect of cognitive aging. The CRI score of the sample ranged from 101 to 136, with an average score of 121 ± 11.

### Data analysis

Trials with RTs faster than 100 and slower than 1500 ms were excluded (1.97%). For each participant, trials above and below 3 *SD* from the mean RT were also excluded (1.06% of total trials). Error trials and trials following an error were not considered in the RT analysis to avoid post-error slowing confounds (Burns, 1965).

Since we were interested in exploring the possible age-related effect specifically on conflict resolution, we applied a logarithmic transformation of RT data in the analysis which was intended to compare the two age groups (see Verhaeghen et al., 2005). This transformation converts proportional effects into additive ones. We thus assume that age-related slowing to be constant across conditions, allowing subsequent ANOVAs to compare younger and older adults across conditions in the absence of group differences in speed. Hence, significant interactions that resist logarithmic transformations can be considered as due to true condition-specific effects. On the contrary, if interactions that were significant before the logarithmic transformation are not significant any more after it, it is possible to assign the effects to general factors such as age-related slowing (Salthouse and Babcock, 1991; Salthouse, 1996).

After logarithmic transformation of raw RT data, we run a 2 × 2 × 2 mixed ANOVA with congruency of trial_*n*_ and congruency of trial_*n* − 1_ as within-subject factors and age group as between-subjects factor. However, we used raw RTs for the analyses conducted within each group (e.g., correlations).

Since the raw accuracy data were not normally distributed, we used the accuracy Stroop effect (measured as the difference between incongruent and congruent trials), which was instead normally distributed, as a dependent variable to perform a 2 × 2 mixed ANOVA with congruency in trial_*n* − 1_ as the within-subjects factor and age group as the between-subjects factor. Moreover, we run a 2 × 2 × 2 mixed ANOVA with congruency of trial_*n*_ and congruency of trial_*n* − 1_ as within-subject factors and age group as between-subjects factor and applied a permutation test to evaluate the interaction between age groups and congruency conditions.

The Mann–Whitney *U*-test was applied, whenever the analysis intended to compare the two age groups across conditions.

In order to explore the amount of interference or facilitation that the presence of two features, either conflicting with each other or not, could produce when responding to a stimulus, we also run the same analysis considering the performance of Direction Only as baseline. Thus, after logarithmic transformation, we subtracted the RTs of Direction Only from those of the other conditions (Position Only, CC, IC, CI, II), whereas, for accuracy, we subtracted the percentage of accuracy for all conditions from that of Direction Only.

Additionally, in order to measure the amount of learning that took place across the three blocks, we run a 2 × 2 × 3 ANOVA (age group × trial_*n*_ congruency × block) on log-RTs, whereas, for the same kind of analysis on accuracy data, given their non-normality, we run a Two Way ANOVA (age group × block) on accuracy Stroop effect. We also conducted correlation analyses between RTs Stroop data and measures of CR, intelligence and years of education. Since these analyses were run separately for each age group, we did not use logarithmic transformations but rather raw data. Furthermore, considering that we collected CR information in the older group only, we wanted to explore the separate contribution of CR and age in the older adults. Hence an ANCOVA was run for the older group only, using age and CRI as predictors.

## Results

### Direction only and position only conditions

**Figure 2:**
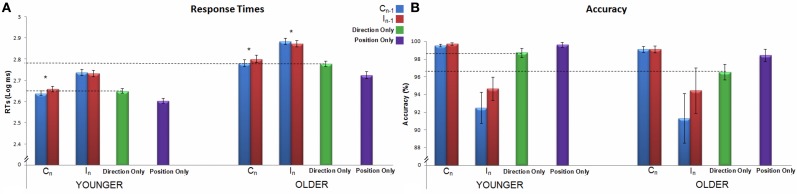
**Response times in ms (Panel A) and accuracy percentage (Panel B) of the spatial Stroop task for both age groups are shown.** On the *x* axis the two spatial Stroop trial_*n*_ conditions are displayed: Congruent (C) and Incongruent (I), ^*^p < 0.05; in addition to the single-feature conditions: Direction Only and Position Only. The colors of the different columns refer to previous trial congruency, as shown in the legend. Dotted lines representing the average performance in Direction Only are drawn to easily compare this condition with the Stroop ones. Error bars represent the standard error of the mean.

Average RTs and accuracy are reported in Table 1. Both younger and older adults showed a better performance in the Position Only condition with respect to the Direction Only one. RTs were indeed significantly shorter in Position Only than in Direction Only for younger ([*t*_(17)_ = 5.29, *p* < 0.001], mean RT: 403 and 447 ms, respectively) and older adults ([*t*_(16)_ = 5.44, *p* < 0.001], mean RT: 534 and 603 ms, respectively) (see Figure 2A). Younger adults showed also a slightly higher accuracy level in Position Only with respect to Direction Only [*t*_(17)_ = 2, *p* = 0.04], whereas this was only a tendency for older adults [*t*_(16)_ = 13, *p* = 0.07] (see Figure 2B). Even after logarithmic transformation of RTs, the older group was slower with respect to the younger one both for Position Only [*t*_(33)_ = −5.79, *p* < 0.001, mean RT: 534 and 403 ms, respectively] and for Direction Only [*t*_(33)_ = −7.64, *p* < 0.001, mean RT: 603 and 447 ms]. Younger and older adults showed a comparable level of general accuracy in both single-feature tasks (Position Only: younger: 99%, older: 98%; Mann–Whitney *U* = 119, *Z* = 1.10, *p* = 0.27; Direction Only: younger: 98%, older: 96%; Mann–Whitney *U* = 105, *Z* = 1.57, *p* = 0.12).

**Table 1:** **Mean RTs and accuracy with respect to conditions and age groups**.

		**CC**	**IC**	**CI**	**II**	**Direction Only**	**Position Only**
Younger	RTs	437 (49)	459 (61)	551 (84)	544 (83)	447 (48)	403 (51)
	Accuracy	99.5 (0.7)	99.7 (0.6)	92.5 (7.5)	94.7 (0.5)	98.6 (2.1)	99.4 (1.2)
Older	RTs	610 (106)	640 (113)	772 (124)	753 (120)	603 (81)	529 (86)
	Accuracy	99.1 (1.4)	99.1 (1.5)	91.0 (11.5)	94.4 (10.6)	96.5 (3.6)	98.1 (2.9)

Standard deviation in parentheses.

### Spatial stroop task

Average RTs and accuracy are reported in Table 1. The group main effect on RTs was significant [*F*_(1, 33)_ = 48.4, *p* < 0.001, η^2^_*p*_ = 0.59], showing that the older group was slower than the younger one. Both the main effects of congruency of trial_*n*_ (i.e., the Stroop effect) and congruency of trial_*n* − 1_ were significant [respectively, *F*_(1, 33)_ = 393.7, *p* < 0.001, η^2^_*p*_ = 0.92; and *F*_(1, 33)_ = 19.7, *p* < 0.001, η^2^_*p*_ = 0.37]. The interaction between trial_*n*_ and trial_*n* − 1_ congruency was also significant [*F*_(1, 33)_ = 80.4, *p* < 0.001, η^2^_*p*_ = 0.71], showing a modulatory effect of trial_*n* − 1_ on the Stroop effect on trial_*n*_ (see Figure 2A). A *post-hoc* Bonferroni comparison showed that both age groups responded faster to CC sequences than to IC ones (*p*s < 0.001); moreover, older adults responded faster to II sequences than to CI ones (*p* < 0.05), while younger adults did not (*p* = 0.16). The interactions involving group × trial_*n*_ congruency, group × trial_*n* − 1_ congruency and the three way group × trial_*n*_ congruency × trial_*n* − 1_ congruency interaction failed to reach significance (for all, *p* > 0.33).

The analysis concerning interference, considering Direction Only as the baseline, confirmed the results reported above. Both the main effects of trial_*n*_ and trial_*n* − 1_ congruency were significant [*F*_(1, 33)_ = 392.8, *p* < 0.001, η^2^_*p*_ = 0.92; and *F*_(1, 33)_ = 20.8, *p* < 0.001, η^2^_*p*_ = 0.39 respectively]. The interaction between these two factors was also significant [*F*_(1, 33)_ = 78.5, *p* < 0.001, η^2^_*p*_ = 0.70]. Neither the main effect of age group [*F*_(1, 33)_ = 1.1, *p* < 0.31] nor the interaction between this factor and trial_*n*_ and trial_*n* − 1_ congruency were significant (*p* = 0.89 and *p* = 0.42, respectively). Thus, independently of age, processing of CC sequences required the same time as the Direction Only condition, whereas interference increased if a congruent trial was preceded by an incongruent one (IC). On the contrary, processing of incongruent trials_*n*_ implied greater interference with respect to Direction Only, and such interference was not modulated by trial_*n* − 1_ congruency.

Regarding accuracy, as specified above, the Stroop effect was used as the dependent variable. The main effect of group was not significant (*p* = 0.848), whereas the main effect of trial_*n* − 1_ congruency was significant [*F*_(1, 33)_ = 8.283, *p* < 0.01, η^2^_*p*_ = 0.20]. The interaction between age group and trial_*n* − 1_ congruency was not significant [*F*_(1, 33)_ = 0.442, *p* = 0.511], suggesting that the modulatory effect of trial_*n* − 1_ on the accuracy Stroop effect of trial_*n*_ is not age-dependent. Due to its non-normal distribution, accuracy was further explored with a permuted mixed model ANOVA in order to check for the group × trial_*n*_ congruency × trial_*n* − 1_ congruency interaction. Such interaction failed to reach significance (*N* = 1000 permutations, *p* = 0.57).

We repeated both these analyses considering Direction Only as the baseline: using the Stroop effect as dependent variable and the mixed model ANOVA coupled with the permutation procedure. The results were perfectly comparable to those of the analyses made using raw data. Results therefore suggest that accuracy does not differ in the two age groups and is modulated by congruency of current trial only (see Figure 2B).

The analysis conducted for exploring the learning rate showed that both age groups increased their speed along the three blocks [main effect of block (*F*_(2, 33)_ = 40.6, *p* < 0.001, η^2^_*p*_ = 0.55)]. On the other hand, the interaction with age and trial_*n*_ congruency was not significant (all *p*s > 0.16, suggesting a non-relevant reduction for the RT Stroop effect throughout the three blocks for both age groups.

Regarding the accuracy Stroop effect, the main effect of block was significant [*F*_(2, 33)_ = 5.70, *p* < 0.005, η^2^_*p*_ = 0.15], whereas neither the main effect of age group nor the interaction between blocks and age group were significant [*p* = 0.749 and *p* = 0.275, respectively], supporting the presence of age-independent learning phenomena for conflict resolution in terms of accuracy.

### Correlational analysis

Table 2 summarizes bivariate correlations among variables.

**Table 2:** **Correlation between measures**.

	**Age**	**Education**	**IQ**	**CRI**
**YOUNGER**				
Education	0.36			
IQ	0.35	0.31		
RTs	0.02	−0.01	−0.09	–
RT Stroop	0.10	−0.07	−0.25	–
RT CI-II	−0.46	−0.03	0.12	–
Accuracy	−0.04	−0.03	0.18	–
Accuracy Stroop	0.03	0.08	−0.22	–
Accuracy II-CI	0.31	0.25	0.20	–
Accuracy Stroop after congruent	0.13	0.14	−0.12	–
Run3–Run1 accuracy Stroop	−0.05	−0.01	−0.03	–
**OLDER**				
Education	−0.05			
IQ	0.05	0.59^*^		
CRI	−0.06	0.81^**^	0.30	
RTs	−0.15	−0.02	−0.26	0.03
RT Stroop	−0.42	−0.12	−0.19	−0.03
RT CI-II	0.07	−0.27	−0.20	−0.52^*^
Accuracy	−0.46	0.45	0.23	0.58^*^
Accuracy Stroop	0.41	−0.44	−0.26	−0.54^*^
Accuracy CI-II	−0.11	−0.37	−0.52^*^	−0.37
Accuracy Stroop after congruent	0.35	−0.47	−0.37	−0.56^*^
Run3–Run1 accuracy Stroop	−0.42	−0.45	−0.33	−0.54^*^

Dashes indicate data not available.

* p < 0.05;

** p < 0.001.

**Figure 3:**
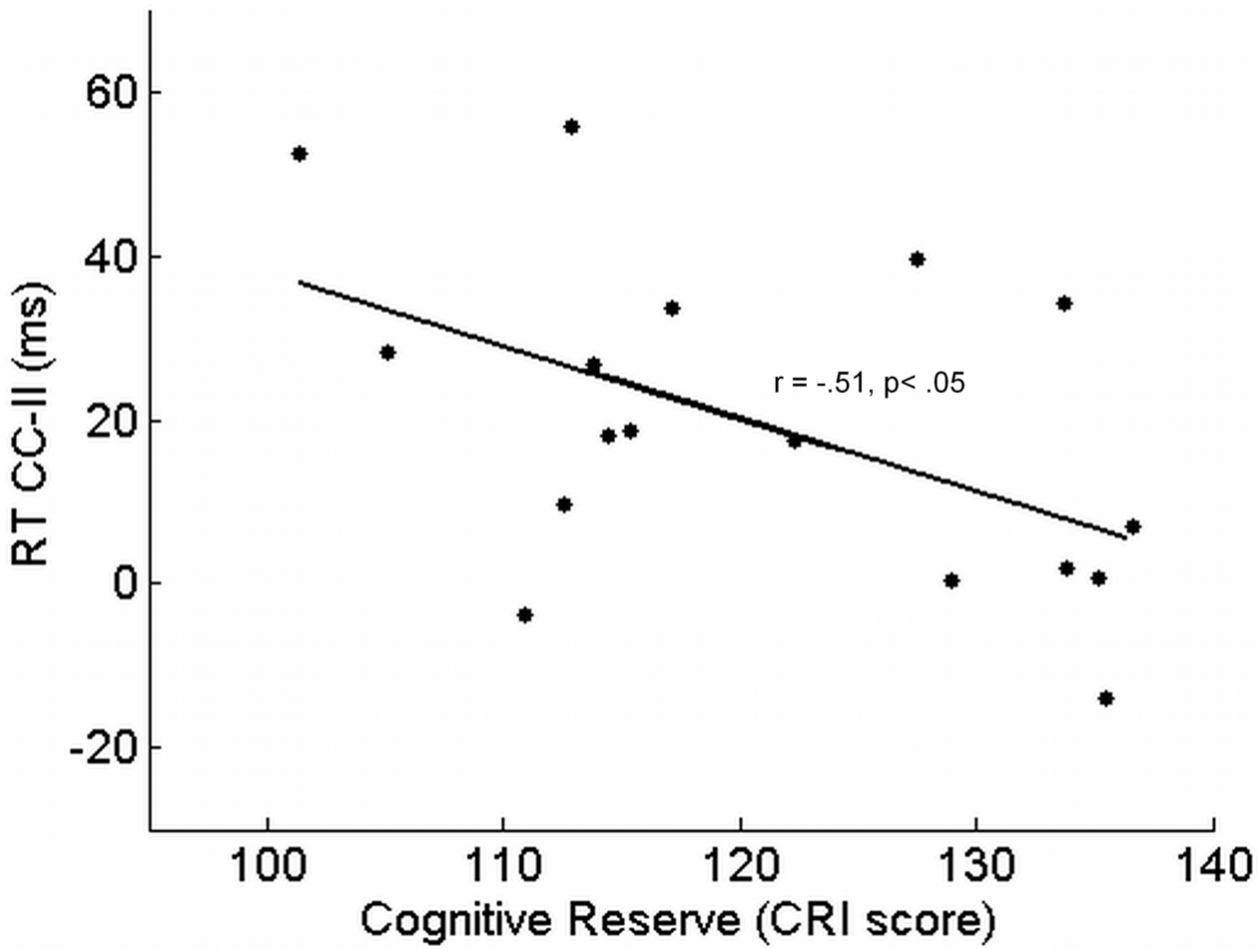
**Response times' difference between congruent-incongruent (CI) and incongruent-incongruent (II) sequences as a function of cognitive reserve, measured as Cognitive Reserve Index, in older adults**.

In older adults, the CRI was associated with a reduction of influence of congruency in trial_*n* − 1_ on conflict resolution in trial_*n*_: the greater the CRI was, the smaller the RT difference between CI and II sequences was [*r*_(15)_ = −0.51, *p* = 0.036, *r*^2^ = 0.26] (see Figure 3).

The ANCOVA considered the RT difference between CI and II sequences as the dependent variable: this analysis revealed that a reduction of such a difference was associated with a high CRI [*F*_(1, 14)_ = 4.91, *p* = 0.043, η^2^_*p*_ = 0.26], whereas the impact of age was not significant [*F*_(1, 14)_ = 0.02, *p* = 0.879].

Learning, measured as the difference between RTs Stroop effects obtained in the first and third blocks, did not correlate in either age group with either age [Younger: *r*_(16)_ = −0.046, *p* = 0.857; Older: *r*_(15)_ = −0.421, *p* = 0.092], or education [Younger: *r*_(16)_ = −0.015, *p* = 0.951; Older: *r*_(15)_ = −0.453, *p* = 0.068], although these correlations showed a trend in the older group.

## Discussion

This study investigated whether aging affects spatial conflict resolution and conflict adaptation, and whether CR, years of education and intelligence may play a compensatory role against age-related deficits in the spatial domain. Many studies reported age-related deficits in verbal conflict resolution, as marked by a substantial increase of verbal Stroop in older adults (e.g., Puccioni and Vallesi, in press; MacLeod, 1991; West and Alain, 2000; West and Moore, 2005), but whether aging also affects spatial conflict resolution has not been investigated as extensively. Some previous studies used spatial Stroop-like tasks with this aim (Van der Lubbe et al., 2002; Bialystok et al., 2004; Proctor et al., 2005; Castel et al., 2007), and found a significant age-related increase of spatial interference. Despite the fact that many authors claimed that priming has a relevant influence in conflict resolution process (e.g., Mayr et al., 2003; Nieuwenhuis et al., 2006; Puccioni and Vallesi, 2012), none of the designs used so far to investigate aging effects on spatial conflict resolution clearly isolated priming effects from pure conflict resolution. Wühr et al., using a Simon-type task, showed the presence of correspondence sequential modulations even after a partial removal of stimulus-response repetitions (Wühr, 2005; Kunde and Wühr, 2006). However, the same authors suggested using a 4-Alternative Forced Choice (4-AFC) version of their task in order to eliminate all possible stimulus-response repetitions (Kunde and Wühr, 2006). Moreover, when investigating aging effects, it is even more important to control for priming contributions, since priming itself is likely to be differentially affected by aging (Connelly et al., 1991; McDowd and Oseas-Kreger, 1991; La Voie et al., 1994; Mayas et al., 2012). We partially circumvented this problem by designing a spatial Stroop task that did not present stimuli with feature repetitions in any two subsequent trials. Thus, priming influences were substantially reduced. For our study, we considered the Stroop effect as a measure of spatial conflict resolution, and therefore calculated it as the difference between the performance on congruent and incongruent trials. Furthermore, we assessed the performance in two single-feature conditions, where the two features composing the stimuli used for the spatial Stroop task, namely direction and position, had to be processed separately. In all the conditions administered (Position Only, Direction Only and Spatial Stroop) older adults were systematically slower with respect to younger adults, although the accuracy level was comparable, confirming the well-known age-related slowing effect (e.g., Rabbitt, 1979; Salthouse, 1985). We confirmed that position-related information is stronger than direction-related information, since the Position Only condition was processed faster and more accurately than the Direction Only condition. In the spatial Stroop task, where both types of information are simultaneously part of the stimulus, participants were asked to respond with respect to direction and to suppress the position information. As expected, we found a significant Stroop effect for both RTs and accuracy. This priming-free spatial Stroop task revealed that older adults were slower than younger controls. Notably, the interaction between age-groups and congruency was not significant either for speed or for accuracy, indicating that the overall spatial Stroop effect did not differ in the two age groups. We also assessed the role of the preceding trial congruency, in order to investigate the influence that it could exert on the spatial Stroop effect. The two age groups equally showed the well-known sequential effects for congruent trials (Botvinick et al., 2001; West and Moore, 2005; Notebaert et al., 2006; Notebaert and Verguts, 2007): RTs were reduced if another congruent trial preceded them, although accuracy was at ceiling. For incongruent trials older adults, but not younger controls, were influenced by the congruency of the trial_*n* − 1_, increasing RTs whenever a congruent one preceded them. This pattern of results was also confirmed by the analyses run on the interference occurring in each age group with respect to its own performance in Direction Only condition. These results are in conflict with the age-related increase of spatial interference that some previous studies reported (Van der Lubbe et al., 2002; Bialystok et al., 2004; Proctor et al., 2005; Castel et al., 2007). However, as we mentioned above, very few previous studies adopted experimental designs which controlled for priming or binding factors; thus they could not properly isolate the aging effects on priming with respect to those on pure conflict resolution. Moreover, some of the previous studies did not apply corrections to account for general slowing.

Despite a non-significant age-related increase of the general Stroop effect, older adults, unlike younger controls, showed an increased difficulty in incongruent trials whenever a congruent trial preceded them. Interestingly, such an age-related modulation negatively correlated with the CR index, but not with intelligence or years of education. Hence our findings suggest that the reduction of specific attention abilities that usually take place in normal aging is attenuated when individuals have a high level of CR. Future samples displaying a wider range of CRI would be useful to investigate all these associations further.

Although some studies used IQ as a measure for cognitive reserve (e.g., Alexander et al., 1997; Albert and Teresi, 1999), other evidence suggested that reserve is more deeply influenced by education and everyday life experiences. Our results support the latter findings, suggesting that intelligence, education and cognitive reserve act separately on building reserve (Rocca et al., 1990; Evans et al., 1993; Stern et al., 1994, 1995; Mortel et al., 1995). Stern's group (Scarmeas et al., 2001; Stern, 2009) reported that in a non-demented older adults' sample, individuals who were more engaged in leisure activities, both intellectual and social, had a reduced risk of developing dementia-independent of the type of activity. Moreover, leisure activities include both physical and mental exercises that have an impact not only on the pure cognitive aspects of reserve, but also on the brain structure, such as an increased brain volume (Stern, 2009). Finding an effect of CR measured with the CRIq but not of education *per se*, suggests that there is something above and beyond the education level that is related to a lower impact of cognitive aging. Therefore, a questionnaire like the one we used to measure cognitive reserve, which considers at the same time education, occupational attainment and leisure activities, seems to be a good proxy for investigating such a complex and still not sharply defined construct. Nevertheless, we think that this instrument could be further improved in the future, particularly by increasing its reliability. Furthermore, considering that participants were administered three blocks, we explored the potential learning effects that took place. Older adults obtained the same advantage as younger controls from practicing, confirming previous findings (e.g., Davidson et al., 2003): both age groups similarly increased their overall speed and the accuracy in conflict resolution (i.e., a comparable reduction of accuracy Stroop effect) over the three blocks.

The participants of the present study also took part in another study (Puccioni and Vallesi, in press), in which they were administered a matched color-word Stroop. This allows us to draw reasonable inferences about the difference that cognitive aging exerts on conflict resolution-related to two different domains: verbal and spatial. In the present study, we showed that spatial Stroop is not affected by aging, whereas the verbal Stroop effect exhibits a marked age-related increase in RTs (but not in accuracy), and roughly an opposite pattern was obtained for sequential effects (see Puccioni and Vallesi, in press). Indeed, in the verbal Stroop task, sequential effects are spared in aging. In both younger and older adults, the RT Stroop effect was nullified after preceding incongruent trials, and sequential effects relative to current congruent trials disappeared, suggesting that they are not due to conflict adaptation, but rather they are likely due to priming (or binding) phenomena. On the contrary, here we show that for the spatial Stroop switching from a congruent to an incongruent trial entails a cost in aging, and sequential effects relative to current congruent trials are present in both age groups, even after controlling for priming contribution. Therefore, our findings concerning verbal and spatial conflict resolution, when considered together, suggest the existence of two different domain-specific mechanisms responsible for conflict resolution rather than a general one. These two separate mechanisms seem to be differentially prone to cognitive aging, since verbal conflict resolution ability is specifically reduced in older adults, whereas spatial conflict resolution seems to suffer from general slowing only. Further studies, possibly with bigger sample sizes, should further test this hypothesis. Another possibility for the different results obtained in the verbal conflict resolution study with respect to the spatial one, could also be that the two tasks require different conflict resolution demands. Unfortunately, since our verbal task did not present the reading only and color naming conditions, it is impossible to directly compare the strength of the dominant condition with respect to the relevant but weaker one, and thus to make a direct comparison with the present spatial task.

It is important to point out that an experimental manipulation of intelligence and CR is not possible. Therefore, a causal relation between CR and a reduced impact of cognitive aging cannot be inferred. Therefore, to adopt longitudinal designs is extremely useful in order to understand whether CR is a reliable predictor of which individuals will be less prone to age-related cognitive decline (e.g., Snowdon et al., 1997; Stern et al., 1999; Riley et al., 2002; Wilson et al., 2002; Salthouse and Ferrer-Caja, 2003). Moreover, such an approach could also clarify if the reduced impact of cognitive aging shown by some individuals is due to a compensatory phenomenon, as suggested by the reserve hypothesis, or rather to pre-existing neural and cognitive characteristics (Nyberg et al., 2012).

In conclusion, the current study suggests that, contrary to verbal conflict resolution, spatial conflict resolution seems to be only marginally affected in healthy cognitive aging. Older adults' performance for spatial conflict resolution and spatial conflict adaptation processes is predominantly affected in terms of a reduction of the overall general speed. Older adults exhibit an age-related deficit in switching from congruent to incongruent conditions, rather than a selective impairment for spatial conflict resolution itself. Nonetheless, this deficit appears to be reduced when the cognitive reserve level is high. It could be that CR plays a compensatory role in maintaining the flexibility of active problem solving in tasks for which solutions cannot be simply derived from prior knowledge or formal education (Horn and Cattell, 1967; Stuart-Hamilton, 1996; Tranter and Koutstaal, 2008), flexibility which is usually prone to an age–related decline. Therefore, our study suggests that older adults whose lives have been characterized by a high level of cognitive reserve might cope better with some aspects of age-related attentional decline.

### Conflict of interest statement

The authors declare that the research was conducted in the absence of any commercial or financial relationships that could be construed as a potential conflict of interest.
